# A Robust Control via a Fuzzy System with PID for the ROV

**DOI:** 10.3390/s23020821

**Published:** 2023-01-10

**Authors:** Junjie Dong, Xingguang Duan

**Affiliations:** School of Mechatronical Engineering, Beijing Institute of Technology, Beijing 100081, China

**Keywords:** type-1 fuzzy system, interval type-2 fuzzy system, ROV

## Abstract

Uncertainty and nonlinearity in the depth control of remotely operated vehicles (ROVs) have been widely studied, especially in complex underwater environments. To improve the motion performance of ROVs and enhance their robustness, the model of ROV depth control in complex water environments was developed. The developed control scheme of interval type-2 fuzzy proportional–integral–derivative control (IT2FPID) is based on proportional–integral–derivative control (PID) and interval type-2 fuzzy logic control (IT2FLC). The performance indicators were used to evaluate the immunity of the controller type to external disturbances. The overshoot of 0.3% and settling time of 7.5 s of IT2FPID seem to be more robust compared to those of type-1 fuzzy proportional–integral–derivative (T1FPID) and PID.

## 1. Introduction

Due to the complexity of the underwater environment, the ROV control system experienced chatter due to interference, which made depth control difficult. Since the ROV depth control has the following characteristics—nonlinearity, temporal fluctuations, and uncertainty—the ROV controller had to possess high stability and robustness [1].

Proportional–integral–derivative (PID) controllers are commonly used for ROV depth control [2,3,4]. Golten introduced a piecewise linearization method to linearize the nonlinear model [5]. Ogata K. improved the traditional PID algorithm by increasing the gain table [6]. In addition, recent research has proposed control algorithms that combine fuzzy algorithms with PID to improve the performance of depth control [7], as well as to control algorithms that combine neural networks with PID [8]. Although PID control is simple, reliable, and has a high degree of accuracy, the use of PID is difficult to optimize and adapt to overcome the nonlinear variables in the ROV environment due to the nonlinear characteristics of the underwater environment. Either a new control algorithm is introduced, or the PID algorithm is combined with other control algorithms to improve the accuracy or scope of the original control algorithm.

With fuzzy control, the effective control of uncertain and highly nonlinear systems can be easily realized. This is especially true for objects for which accurate mathematical models are difficult to construct [9]. Although the interval type-2 fuzzy logic system (IT2FLS) has been further developed, the type-1 fuzzy logic system (T1FLS) is still widely used, e.g., single-input fuzzy control for unmanned underwater vehicles (UUVs) [10], ROV navigation control [11], ROV trajectory control [12], exoskeleton robot system [13], and borehole backpressure control [14]. The wide application of type-1 fuzzy logic controllers (T1FLC) is due to their usefulness in the presence of uncertainties. However, the control performance of T1FLC deteriorates significantly when various dynamic uncertainties occur. Other controllers are also applied to underwater robots; reference [15] uses a sliding mode control to realize ROV trajectory tracking, reference [16] uses an event-driven controller to set up ROV attitude stabilization system, and reference [17] introduces a sliding mode backstepping control to realize ROV control.

With the development of IT2FLS, IT2FLS exhibits more advantages than T1FLS in modeling uncertainty and can directly incorporate uncertainty into fuzzy sets. This built-in uncertainty improves the flexibility of the system in dealing with noise and unknown data. To solve the stability problem of the nonlinear model in [18], an IT-2 fuzzy controller was designed. In reference [19], the asymptotic stability of IT2FLS was studied, based on extended discrete functions in the case of mismatched membership functions. Li [20] used the IT-2 fuzzy model method to discuss the nonlinear network control problem of packet loss. The IT-2 fuzzy model-based method in reference [21] is used to study the robust fault detection problem for a class of nonlinear stochastic systems. In addition, fuzzy tracking control of real-time systems has gained attention in recent years [22,23,24,25,26]. Liu [27] proposed a type-2 FLC (T2FLC) for bipedal robots to ensure gait stability and achieve robust control performance. Unfortunately, no systematic method has yet been introduced to identify the fuzzy parameters MF (member function) required to satisfy the stability criteria. Juang and Hsu [28] proposed an optimized T2FLC for wall tracking of mobile robots with noisy readings in uncertain environments. Although good control performance can be achieved with an optimized T2FLC, this method still uses an offline learning phase, which prevents the controller from achieving its full performance in a changing environment. Lin [29] proposed an adaptive interval T2FLS (AIT2FLS) for tracking control of non-linear multi-input multi-output (MIMO) systems to handle training data corrupted by noise or control uncertainties. However, the proposed AIT2FLS control scheme uses a back-propagation gradient-descent method to adapt to fuzzy parameters, which cannot guarantee the stability of the system and is not suitable for online adaptive control. Lam and Seneviratne [30] proposed an interval-type-2 Takagi–Sugeno–Kang fuzzy logic system (IT2TSKFLS) control scheme for nonlinear systems with uncertain parameters. Moreover, Biglarbegian [31] introduced a simple reasoning mechanism for designing IT2TSKFLS for real-time control applications.

Based on these findings, a novel robust adaptive interval type-2 fuzzy PID (IT2FPID) is proposed for depth control in remotely operated vehicles (ROVs). Considering the viscosity of the underwater environment and the accuracy of the control model, a control algorithm based on the interval type-2 fuzzy law (AIT2F) is proposed, which is more suitable than the conventional fuzzy algorithm, has better performance in solving uncertainties, and shows greater robustness.

The contribution of this paper is twofold. (1) An interval method type-2 fuzzy proportional integral derivative (IT2FPID) combining IT2FLS and PID is proposed to achieve ROV depth control, and it is shown that this method is superior to type-1 fuzzy proportional integral derivative (T1FPID) and PID in the application of robot depth control. (2) In the presence of different noises, a comparative simulation of the noise immunity of the type reduction method (TR) was performed, and the robustness of the different methods to different noises was evaluated.

The rest of this article is organized as follows. Section 2 discusses the dynamic modeling of ROV depth control. Section 3 provides a systematic explanation of IT2FLS. Section 4 analyses the simulation results of the IT2FPID controller, comparing it with the T1FPID and PID controller, showing the advantages of using IT2FPID, and Section 5 provides some conclusions and comments on the possibilities of future work.

## 2. Problem Representation

The complete spatial motion of the ROV should be described in the fixed coordinate system and the ROV body reference system. The specific forms are as follows [32].

The origin of the fixed coordinate system is *E*, the x axis is in the horizontal plane, and the y, z axis establishes a vertical coordinate system, according to the right-hand rule.

**Figure 1:**
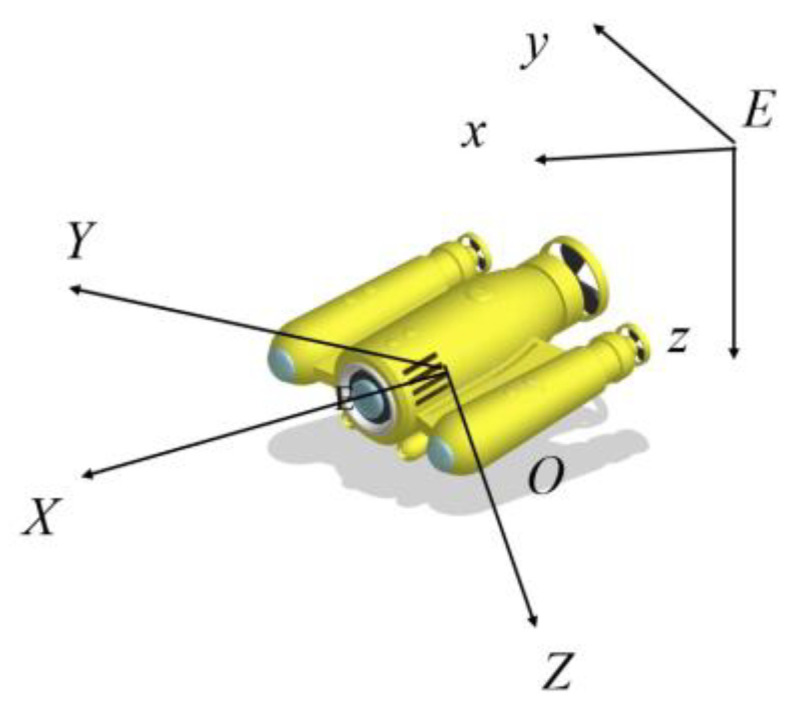
Coordinate diagram.

The origin of the ROV body reference system is *O*, which is at the geometric center, the OX *X* axis is the same direction as the positive symmetry axis of the underwater vehicle body structure, the *Y* axis is parallel to the baseline of the underwater vehicle body structure and perpendicular to the *X* axis, and *Z* is perpendicular to the $O_{XY}$ plane, as shown in Figure 1.

The definitions of speed, angular velocity, and external force in the ROV body reference system are shown in Table 1.

### 2.1. Kinematics and Problem Formulation

The motion of the ROV is essentially a six degrees of freedom spatial motion, which can be represented by translational motion along three axes and rotational motion around three axes. This paper only considers the translational governing equation in the *z* direction, that is, the governing equation in the depth direction.

According to Newton’s second law, the expression of the force received by the underwater robot is as follows:

(1) $$m\frac{dV_{G}}{dt}=F$$

In order to satisfy the generality of the ROV motion, in the fixed system, the speed of the center of gravity $V_{G}$ can be divided into the following:

(2) $$V_{G}=V+\Omega \times R_{G}$$

where *V* is the speed of the origin of the motion system in the fixed system; Ω is the rotational angular velocity of the motion system; $R_{G}$ is the center of gravity to the coordinate origin point distance; and $\Omega \times R_{G}$ is the convective velocity. Now, the vector expression of the center of gravity acceleration is as follows:

(3) $$\overset{\cdot }{V_{G}}=\frac{dV_{G}}{dt}=\frac{d(V+\Omega \times R_{G})}{dt}=\overset{\cdot }{V}+\Omega \times V+\overset{\cdot }{\Omega }\times R_{G}+\Omega \times (\Omega \times R_{G})$$

where $\dot{V}$ is the acceleration of the origin under the dynamic system, and $\dot{\Omega }$ is the angular acceleration.

The above formula is written in the form of force components in three directions:

(4) $$\left\{\begin{array}{l} X=m\left[\overset{\cdot }{u}-vr+wq-x_{G}(q^{2}+r^{2})+y_{G}(pq-\overset{\cdot }{r})+z_{G}(pr+\overset{\cdot }{q})\right]\\ Y=m\left[\overset{\cdot }{v}-wp+ur-y_{G}(p^{2}+r^{2})+z_{G}(qr-\overset{\cdot }{p})+x_{G}(pq+\overset{\cdot }{r})\right]\\ Z=m\left[\overset{\cdot }{w}-uq+vp-z_{G}(q^{2}+p^{2})+x_{G}(pr-\overset{\cdot }{q})+y_{G}(qr+\overset{\cdot }{p})\right] \end{array}\right.$$

where $x_{G\;}$ $y_{G}$ $z_{G}$ is the position of the center of gravity of the robot in the ROV body reference system. this paper only studies the depth control, which is the motion in the *z* direction, that is:

(5) $$Z=m\left[\overset{\cdot }{w}-uq+vp-z_{G}(q^{2}+p^{2})+x_{G}(pr-\overset{\cdot }{q})+y_{G}(qr+\overset{\cdot }{p})\right]$$

### 2.2. Dynamics Model

The main force that ROV receives during underwater movement can be roughly divided into the following: hydrodynamic force, driving force, gravity, buoyancy, and interference force. The resultant force and moment experienced by the ROV can be expressed as follows:

(6) $$F=F_{i}+F_{v}+F_{f}+F_{G}+F_{h}+F_{th}$$

To clarify, $F_{i}$ is the inertial hydrodynamic force, $F_{v}$ is the viscous hydrodynamic force, $F_{f}$ is the driving force, $F_{G}$ is static, $F_{h}$ is the tension of the umbilical cable, and $F_{th}$ is the external uncertain force. Assuming that the ROV is small enough and a zero-buoyancy cable is used, the cable resistance and environmental interference can be ignored.

The resultant force component in the *z* direction is expressed as:

(7) $$Z=Z_{i}+Z_{v}+Z_{f}+Z_{G}+Z_{h}+Z_{th}$$

When fully drained, the gravity and buoyancy should essentially be balanced, but at the same time, a small positive buoyancy should be left to ensure the safety of the ROV when it fails. Assuming that the coordinates of the center of gravity of the underwater robot are $G_{0}\left(x_{G},y_{G},z_{G}\right)$, and the coordinates of the center of buoyancy are $P_{0}\left(x_{P},y_{P},z_{P}\right)$, Then the effect of static force on ROV is [33]:

(8) $$Z_{G}=(G_{0}-P_{0})\cos\theta \cos\varphi$$

where θ  is the angle of heel, and φ is the angle of pitch. The specific form of hydrodynamics is difficult to express with functions. The hydrodynamics experienced by the ROV under the action of a certain flow field can be divided into inertial hydrodynamic forces and viscous hydrodynamic forces. The specific size is related to the ROV’s state of motion, and its overall form can be expressed in functional form:

(9) $$F_{F}=f(V,\overset{\cdot }{V,}\Omega ,\overset{\cdot }{\Omega })=f(u,v,w,\overset{\cdot }{u},\overset{\cdot }{v},\overset{\cdot }{w},p,q,r,\overset{\cdot }{p},\overset{\cdot }{q},\overset{\cdot }{r})$$

Hydrodynamics is divided into inertial hydrodynamics and viscous hydrodynamics. The hydrodynamic force has a linear relationship with acceleration and angular acceleration, and the proportional coefficient $\lambda_{ij}$ of the two is called the additional mass.

(10) $$F_{j}=-\sum_{j=1}^{6}\lambda_{ij}\overset{\cdot }{U_{i}}$$

where $F_{j}={\left(X_{j},Y_{j},Z_{j},K_{j},M_{j},N_{j}\right)}^{T}$; $U_{i}={\left(\dot{u},\dot{v,}\dot{w},\dot{p,}\dot{q,}\dot{r}\right)}^{T}$.

The hydrodynamic force in the vertical plane can be expressed as a multivariate function of velocity, acceleration, angular velocity, and angular acceleration. Therefore, the inertial hydrodynamic formula in the *z* direction is:

(11) $$Z_{i}=-\lambda_{33}\overset{\cdot }{w}-\lambda_{36}\overset{\cdot }{q}-\lambda_{24}p^{2}+\lambda_{11}uq-\lambda_{22}vp-\lambda_{26}pr$$

Usually, the multivariate function Taylor expansion method is used to solve the calculation. When analyzing the viscous hydrodynamic force, the tiny high-order terms are ignored, and only the quadratic terms are retained. According to the symmetrical characteristics of the main structure of the ROV, the calculation formula of the viscous hydrodynamic force in the *z* direction is as follows:

(12) $$\begin{array}{l} Z_{v}=Z_{w}\overset{\cdot }{w}+Z_{q}\overset{\cdot }{q}+Z_{w}w+Z_{\left|w\right|}\left|w\right|+Z_{q}q\\ +Z_{w\left|w\right|}w\left|w\right|+Z_{ww}w^{2}+Z_{w\left|q\right|}w\left|q\right|+Z_{q\left|q\right|}q\left|q\right| \end{array}$$

The main performance is that the umbilical cable is pulled on the horizontal and vertical planes during the movement. Under the Wilson model [34], the force of the umbilical cable on the vertical plane under the action of the sea current is as follows:

(13) $$Z_{h}=\frac{1}{2}\rho C_{n}u_{w}^{2}\sin\psi \sin\left|\psi \right|$$

where ρ is the density of sea water, $C_{n}$ is the normal force coefficients, and the value is 0.04 $u_{w}.$ The speed of water flow relative to the robot is opposite to the speed of robot movement, and ψ is the angle between the direction of the incoming flow and the cable.

Assuming that the angle between the incoming flow direction and the cable is 0°, and *d* is 0.007 mm, it can simplify the above formula to obtain the following:

(14) $$Z_{h}=-3.52u_{w}^{2}$$

Since this paper only considers the motion of the vertical plane, the kinematics analysis of the vertical plane only involves u,$\dot{u}$, w, $\dot{w}$,$\;q,\;\dot{q}$, ignoring other motion parameters, and the influence of water $u_{w}=0$, Equation (7) can be obtained as follows:

(15) $$\begin{array}{l} m\left(\overset{\cdot }{w}-uq\right)=Z_{f}-\lambda_{31}\overset{\cdot }{u}-\lambda_{33}\overset{\cdot }{w}+Z_{w}w+Z_{\left|w\right|}\left|w\right|+Z_{q}q\\ +Z_{w\left|w\right|}w\left|w\right|+Z_{\left|ww\right|}w^{2}+Z_{w\left|q\right|}w\left|q\right|+Z_{q\left|q\right|}q\left|q\right|+\left(G-P\right)\cos\theta \end{array}$$

thus, no movement in other directions during snorkeling, as with $u=\dot{u}=q=\dot{q}=\theta =0$ and the equation that can be obtained is as follows:

(16) $$m\overset{\cdot }{w}=Z_{f}-\lambda_{33}\overset{\cdot }{w}+Z_{w}w+Z_{\left|w\right|}\left|w\right|+Z_{w\left|w\right|}w\left|w\right|+Z_{\left|ww\right|}w^{2}+\left(G-P\right)$$

Additionally, $\dot{\zeta }=w$, $\ddot{\zeta }=\dot{w}$ ignores the small non-linear hydrodynamic coefficients and the directional coupled hydrodynamic coefficients $Z_{\left|w\right|}$, $Z_{w\left|w\right|}$, $Z_{\left|ww\right|}$ and positive buoyancy G−P=ΔG, consequently, the final depth control motion equation can be written as:

(17) $$\left(m+\lambda_{33}\right)\overset{\cdot \cdot }{\zeta }=Z_{f}+Z_{w}\overset{\cdot }{\zeta }+\left(G-P\right)$$

$\lambda_{33}$, $Z_{W}$ are dimensionless hydrodynamic coefficients.

Since the rotation characteristic of the motor is the inertia link, the motor model can be approximated as a typical first-order linear link to manage:

(18) $$H_{EM}\left(s\right)=\frac{K_{E}}{T_{E}s+1}$$

Among them, $K_{E}$ is the time constant of the motor, and $T_{E}$ is the amplification factor of the motor. The specific value can be estimated from the motor structure parameters the and experiments.

The thrust generated by the propeller is proportional to the square of its speed, which is a nonlinear relationship. In order to obtain the linearized model, it must be linearized. The transfer function of the propeller is:

(19) $$H_{p}\left(s\right)=C$$

Combining Equations (17)–(19) can obtain the final dynamic equation of depth.

The parameters of the ROV are shown in Table 2.

## 3. Fuzzy Controller Design

**Figure 2:**
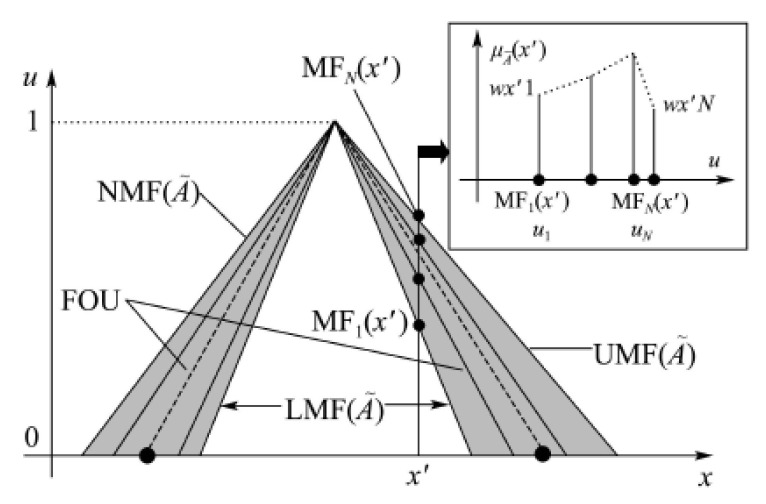
Type-2 fuzzy set in a two-dimensional plane.

Let $\tilde{A}$ be a certain type-2 fuzzy set (T2-FS), x∈X is the primary variable representing the actual physical quantity, and $u\in J_{x}$ is the secondary variable, where $J_{x}\subseteq \left[0,1\right]$ is the primary membership. T2-FS can be regarded as a surface in a three-dimensional space $X\times \left[0,\;1\right]\times \left[0,\;1\right]$, which needs to be represented by a three-dimensional membership function, and Figure 2 is a simplified two-dimensional representation. As shown in the Figure 2, for any $x^{\prime }\in X$, then $J_{x^{\prime }}=\left[MF_{1}\left(x^{\prime }\right),MF_{N}\left(x^{\prime }\right)\right]$, where *N* is the number of discrete points of $J_{x^{\prime }}$. In this way, $\tilde{A}$ can be expressed as follows:

(20) $$\tilde{A}=\int_{x\in X}\left(\int_{u\in J_{x}}\mu_{\tilde{A}}\left(x,u\right)/u\right)/x$$

The secondary membership function is the vertical slice in Figure 2, which can be expressed as:

(21) $$\mu_{\tilde{A}}\left(x^{\prime }\right)=\int_{u\in J_{x^{\prime }}}\mu_{\tilde{A}}\left(x^{\prime },u\right)/u$$

The secondary grade is:

(22) $$f_{x^{\prime }}\left(u_{i}\right)=\mu_{\tilde{A}}\left(x^{\prime },u_{i}\right)=W_{x^{\prime }i},i=1,\ldots ,N$$

T2-FS with a secondary grade of 1 is defined as an interval type-II fuzzy set (interval T2-FS, IT2-FS).

The footprint of uncertainty (FOU), which can be expressed as:

(23) $$FOU\left(\tilde{A}\right)=\int_{x\in X}J_{x}/x$$

where the upper bound is UMF $\tilde{\left(A\right)}$, and the lower bound is LMF $\tilde{\left(A\right)}$. Any T1-FS in the *FOU* is defined as an embedded type-I fuzzy set (ET1-FS), which is expressed as follows:

(24) $$A_{e}=\int_{x\in X}u/x,u\in J_{x}$$

It can be seen that UMF $\tilde{\left(A\right)}$ and LMF $\tilde{\left(A\right)}$ also belong to ET1-FS. The embedded T2-FS (ET2-FS) is defined as ET1-FS with sub-membership values, which can be expressed as:

(25) $$\tilde{A_{e}}=\int_{x\in X}\left(\mu_{\tilde{A}}\left(x,u\right)/u\right)/x,u\in J_{x}$$

**Figure 3:**
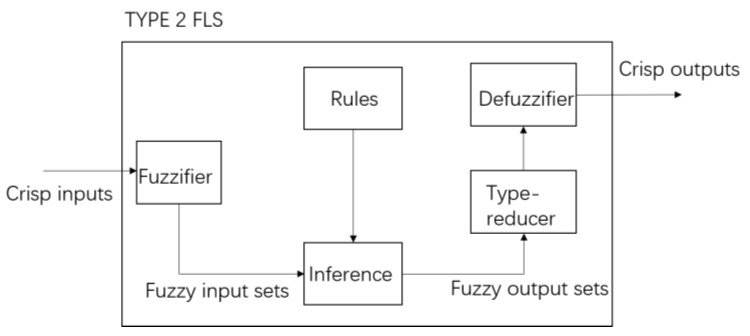
Block diagram describing an IT2FLS.

As long as the membership function of the front or back part of the fuzzy rule contains T2-FS, the corresponding fuzzy system is called T2-FLS. As shown in Figure 3, the difference between T2-FLS and T1-FLS is that the output processing is added in the type-reduction (TR) part. The fuzzy inference output in T2-FLS is T2-FS, so it needs to be reduced to the type-I TR set first, and then defuzzification can be performed to obtain a clear output.

Consider the IT2FLS with a single input and a single output, where the input is $x_{i}\in X_{i}\subset R$ and the output is y∈Y⊂R, assuming that IT2FLS contains the following M fuzzy rules:

$$R^{l}:\;\mathrm{IF}\;x_{1}\;\mathrm{is}\;F_{1}^{l}\;\mathrm{and}\ldots \mathrm{and}\;x_{p}\;\mathrm{is}\;F_{p}^{l},\;\mathrm{then}\;y\;\mathrm{is}\;G^{l},\;\mathrm{where}\;l=1,\ldots ,M$$

TR methods mostly include the Karnik–Mendel Algorithm (KM) [35], the Wu–Mendel Uncertainty Bound Method (WM) [36], the Nie–Tan Method (NT) [37], the Begian–Melek–Mendel Method (BMM) [38], and some others. This paper will compare these algorithms later in the simulation section, but we now introduce the KM algorithm:

(26) $$C_{\tilde{B}}=[c_{l}\left(\tilde{B}\right),c_{r}\left(\tilde{B}\right)]$$

(27) $$c_{l}\left(L\right)=\frac{\sum_{i=1}^{L}y_{i}{\bar{\mu }}_{\tilde{B}}\left(y_{i}\right)+\sum_{i=L+1}^{N}y_{i}{\underline{\mu }}_{\tilde{B}}\left(y_{i}\right)}{\sum_{i=1}^{L}{\bar{\mu }}_{\tilde{B}}\left(y_{i}\right)+\sum_{i=L+1}^{N}{\underline{\mu }}_{\tilde{B}}\left(y_{i}\right)}$$

(28) $$c_{r}\left(R\right)=\frac{\sum_{i=1}^{R}y_{i}{\underline{\mu }}_{\tilde{B}}\left(y_{i}\right)+\sum_{i=R+1}^{N}y_{i}{\bar{\mu }}_{\tilde{B}}\left(y_{i}\right)}{\sum_{i=1}^{R}{\underline{\mu }}_{\tilde{B}}\left(y_{i}\right)+\sum_{i=R+1}^{N}{\bar{\mu }}_{\tilde{B}}\left(y_{i}\right)}$$

among them, $y_{i}$ represents discrete points on the output universe *Y*, ${\underline{\mu }}_{\tilde{B}}$ and ${\bar{\mu }}_{\tilde{B}}$, respectively, represent the LMF and UMF of $\tilde{B}$. The switching points *L* and *R* are the discrete point numbers that allow $c_{l}$ to obtain the minimum value and $c_{r}$ to obtain the maximum value. In this way, the TR of IT2-FLS based on the KM algorithm can be expressed as follows:

(29) $$y_{l}\left(x\right)=\underset{\forall f^{l}\in [\underline{f^{l}},\bar{f^{l}}]}{\min}[\sum_{l=1}^{M}y_{l}^{l}f^{l}/\sum_{l=1}^{M}f^{l}]$$

(30) $$y_{r}\left(x\right)=\underset{\forall f^{l}\in [\underline{f^{l}},\bar{f^{l}}]}{\max}[\sum_{l=1}^{M}y_{r}^{l}f^{l}/\sum_{l=1}^{M}f^{l}]$$

among them, $\left[y_{l}^{l},y_{r}^{l}\right]$ is the fuzzy rule consequence ${\bar{G}}^{l}$ centroid interval obtained using the KM algorithm, and $\left[{\underline{f}}^{l},{\bar{f}}^{l}\right]$ is the interval activation set. It is solved by Equation (26). In this way, the defuzzification of IT2-FLS can be expressed as:

(31) $$y=\left(y_{l}\left(x\right)+y_{r}\left(x\right)\right)/2$$

**Figure 4:**
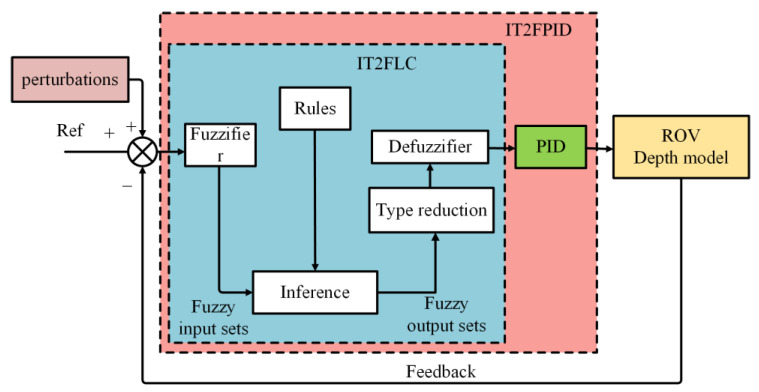
Control schemes for the interval type-2 fuzzy proportional integral derivative (IT2FPID).

Figure 4 presents an overview of the IT2FPID architecture in ROV depth control.

The figure above shows the structure of the entire control system. First, the difference between the reference and the feedback is input to the IT2FLC controller, which includes the fuzzifier, inference, rules, type reduction, and defuzzifier. Then, the output is provided to the PID controller in an incremental manner. Among these, an important factor that determines the robustness of IT2FPID is type reduction, so choosing a type reduction with good performance is a key step in improving the robustness of the system.

## 4. Simulation Results and Discussion

In this section, we use two cases to prove the feasibility and robustness of the proposed IT2FPID using MATLAB/Simulink.

### 4.1. Simulation with Perturbations

**Figure 5:**
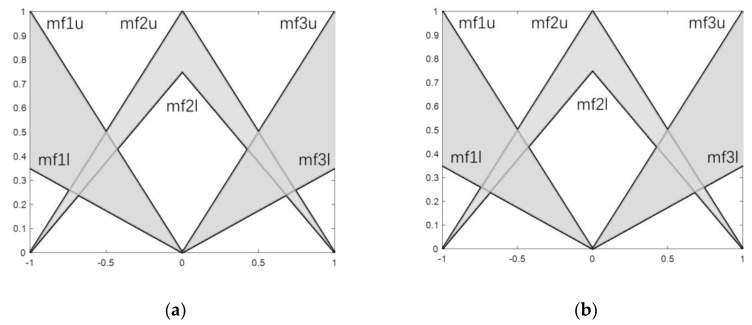
Membership function distribution for (**a**) e and (**b**) $\dot{e}$ input with IT2FS.

The ROV was modeled with an IT2FPID controller. The depth error and the derivative of the depth error are the input, and the torque is the output. The membership function is triangular for the Negative (N), Negative Big (NB), Negative Medium (NM), Positive (P), Positive Big (PB), Positive Medium (PM), and Zero (Z) linguistics terms. Table 3 shows a set of fuzzy rules. Figure 5 shows the IT2FC membership function sets of e and $\dot{e}$, respectively. The difference between the actual value detected by the sensor and the set point can be used to determine the value of the deviation and the rate of change in the deviation at each instant. It is a continuous value that must be converted to a discrete quantity required for input to the controller in the process of discretizing the input domain and converting it into the speech value of the corresponding fuzzy set on the discourse domain, that is, fuzzification. However, this article does not involve experiments, and does not require discretization, which is needed in actual experiments.

Both inputs and outputs are in the range of [−3, 3], based on the literature review and previous experience. However, in order to better verify the performance of the small open-frame underwater robot controller, the two inputs [−1, 1] are chosen. Experimentation was performed with external interference—pulse generated noise. The amplitude and period of pulse generated noise, width, and phase delay were 0.1, 1, 10, and 0, respectively. The simulation time of the system was 40 s. The excitation function is a step response function, where step time, initial value, final value, and sample time are 1, 0, 1, and 0, respectively. The entire controller has two inputs—one is the depth error, and the other is the derivative of the depth error—and the output is the torque of the motor. The simulation of ROV is the depth trajectory tracking in the depth *z* direction, and we also added the interference of the pulse signal. The details of the interference signal have been given above.

The experimental standard is a series of performance indicators often used in control [39,40,41], such as integral time absolute error (*ITAE*), integral time square error (*ITSE*), integral absolute error (*IAE*), integral square value (*ISV*), and integral square error (*ISE*); the formula is as follows:

(32) $$ITAE=\int_{0}^{\infty }\left|e\left(t\right)\right|tdt$$

(33) $$ITSE=\int_{0}^{\infty }e^{2}\left(t\right)tdt$$

(34) $$IAE=\int_{0}^{\infty }\left|e\left(t\right)\right|dt$$

(35) $$ISE=\int_{0}^{\infty }e^{2}\left(t\right)dt$$

(36) $$ISV=\int_{0}^{\infty }x^{2}\left(t\right)dt$$

The results in Table 4. show the performance of the different cases under the four TR methods, calculated with and without noise.

To better understand the results discussed in this section, several points need to be considered. First, the KM method was used to obtain the optimal PID parameters through the trial and error method so that the IT2FPID achieved its ideal performance. Based on this, the TR method was replaced, and the performance of the TR of the four methods was compared. The parameters of the IT2FPID controller are K_P_ = 15, K_I_ = 0.01, and K_D_ = 80. K_P,_ K_I,_ K_D_ correspond to the proportional factor, the integral factor, and the derivative factor, respectively.

Second, the results of the IT2FPID control rules and the membership function were summarized, based on experience.

**Figure 6:**
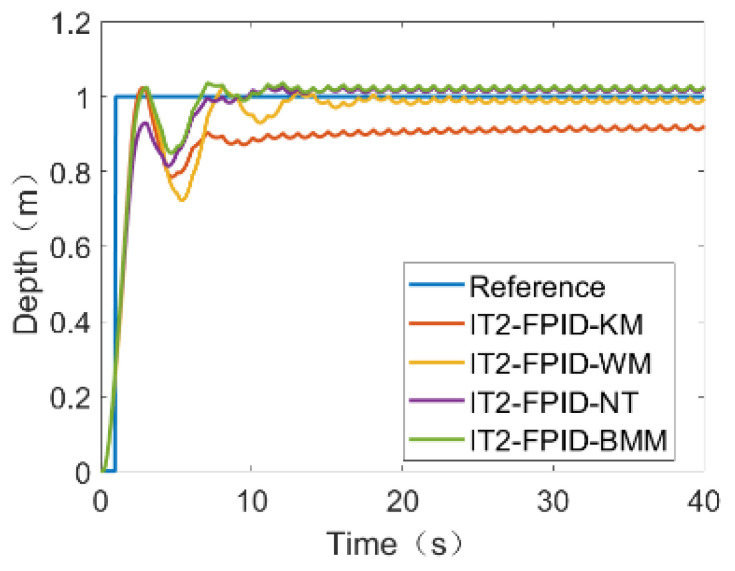
The simulation results of impulse noise.

In the case of external disturbance, the results are shown in Figure 6. After comparing the four TR methods, the smaller the value, the better the control performance. It was found that NT showed the best performance, followed by BMM.

We see that the algorithm KM has a larger steady-state error in pulsating noise than the other three, and there is a large fluctuation at about 7s for the four, which is due to the fact that there is a large amplitude interference at this time. From the Figure 6, it can be seen that the algorithm NT reaches the reference value quickly and exhibits the least overshoot.

To compare the performance differences of the four methods of TR in more detail, a comparative simulation without noise was performed.

We will compare the performances of the control systems with respect to the values of the following: settling time (Ts), overshoot (OS%), and average computational times (CTs). For a fair comparison and to show the effect of the TR method on the controller performance, we will set and fix the scaling factors of IT2FPID as K_P_ = 15, K_I_ = 0.01, and K_D_ = 80 [42,43].

**Figure 7:**
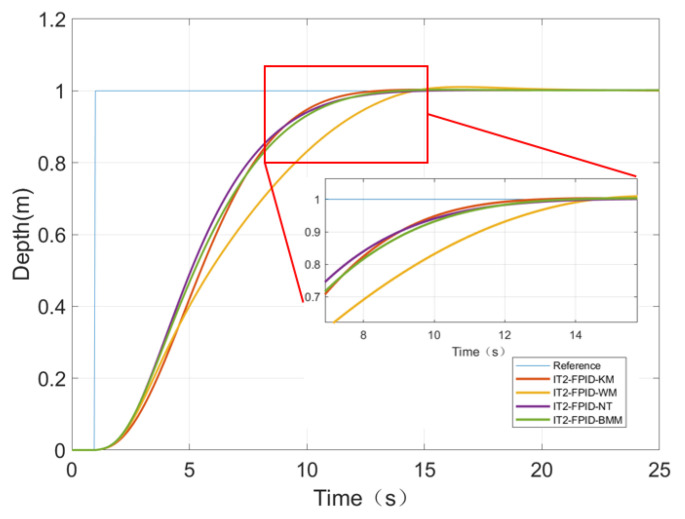
Comparison results of simulation without noise.

The simulation results are shown in Figure 7. The corresponding performance measures are tabulated in Table 5. It can be clearly seen that the Ts of the four TR methods are the same. On the other hand, IT2FPID can reduce the OS% to close to zero. Moreover, although the TR defuzzification method is a structural parameter, it must be determined according to the design criteria. For this specific simulation study, we can see that the OS and CTs values of the NT method are the smallest among the four methods. However, in order to be able to generalize the results of these observations, extensive comparative simulations and real-time research must be conducted.

### 4.2. Simulation without Perturbations

Various loads were then used to test the effectiveness of the proposed scheme. The results of the above IT2FPID control method were compared with PID and T1FPID. The relevant parameters of the three different control methods were determined by the trial and error method, allowing all three controllers to achieve optimal performance.

The parameters of the IT2FPID, PID, and T1FPID controllers are as shown in Table 6.

**Figure 8:**
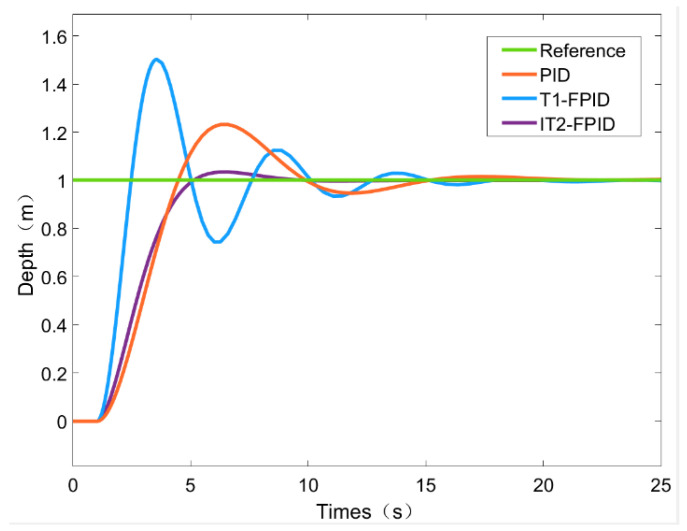
Step response results regarding depth.

To illustrate the performance of each controller, Figure 8 shows the depth control performed by the ROV using an IT2FPID and two different controller types. For example, Figure 8 shows the performance of the fuzzy behavior for depth control based on an IT2FPD controller and a T1FPID controller, respectively. From the figure, it can be seen that the time required to reach a stable state is different. This is a natural response due to the different performances of each controller. As pointed out in reference [44], the inclusion of higher order fuzzy controllers not only allows for more degrees of freedom in the fuzzy sets, but also improves system stability by including better handling uncertainty. Recent advances in the development of higher order fuzzy logic controllers, such as general type-2 fuzzy controllers (GT2 FLCs) [45] and interval type-3 fuzzy control controllers (IT3 FLCs), have been shown to outperform conventional fuzzy controllers by better handling dynamic disturbances and actuator nonlinearities [46]. Because the upper and lower parts of the FOU of IT3 FLCs are not constant, and the secondary MF is interval type 2. In GT2 and IT2 FLCs, T1 provides fuzzy sets and crisp numbers, respectively.

The effectiveness of the proposed IT2FPID has been clearly demonstrated by the simulation results, and the proposed control method is very robust, even in the absence of noise.

From Table 7, we can see that these three methods of underwater depth control can be achieved with the ROV, but they are different in terms of their robustness and control performance. The OS% of IT2FPID is the smallest among these three control methods, followed by that of T1FPID and PID. The steady-state error of all control methods eventually approaches zero. The smallest overshoot is for IT2FPID, but the best response time is for T1FPID. Considering the superior overshoot performance of the IT2FPID control scheme, we believe that this sacrifice of control response speed is acceptable.

Briefly, the superiority of IT2FPID in overcoming the uncertainty and robustness of ROV underwater depth control is proven in this simulation.

## 5. Conclusions

In this paper, an IT2FPID control method for achieving depth performance with an ROV is proposed. In ROV underwater depth control, the controller response speed, control accuracy, and robustness are important indicators due to the characteristics of the underwater environment. The proposed IT2FPID has advantages over T1FPID and PID for these three indicators. The main reason for using IT2FPID is that IT2FPID has higher tolerance to uncertain systems. The conclusion of this paper is that using IT2FPID is a good choice when the problem has a high level of uncertainty. In other words, the IT2FPID can better model the uncertainty because it has more degrees of freedom than PID and T1FPID. Although the IT2FPID offers advantages in terms of the stability and robustness of the depth control, it is clear that the start-up speed of this method, which may be related to the complexity of the algorithm, is not one of its strengths. In the future, it will be necessary to further investigate the response speed of IT2FPID to the excitation in regards to depth control.

## Figures and Tables

**Table 1:** Motion parameters and ROV body reference system components of force.

Items	X-Axis	Y-Axis	Z-Axis
velocity *V*	*u*	*v*	*w*
angular velocity *Ω*	*p*	*q*	*r*
force *F*	*X*	*Y*	*Z*

**Table 2:** ROV parameters.

Items	Values	Items	Values
ROV length (m)	0.42	M (kg)	7.53
ROV width (m)	0.26	*Z_w_*	−0.422
ROV height (m)	0.2	λ_33_	3.251

**Table 3:** Fuzzy rules used by the FC.

e	$\dot{e}$	Out
N	N	NB
N	Z	NM
N	P	Z
Z	N	NM
Z	Z	Z
Z	P	NM
P	N	Z
P	Z	PM
P	P	PB

**Table 4:** Performance index results when inserting noise perturbations.

Type of Noise	Performance Index	KM	BMM	WM	NT
No noise	ITAE	187.60	70.82	87.08	76.09
	ITSE	30.77	13.69	20.26	15.96
	IAE	4.69	1.77	2.17	1.90
	ISE	0.76	0.34	0.50	0.39
	ISV	32.64	15.12	21.40	16.77
Pulse noise	ITAE	201.95	191.8	226.86	187.30
	ITSE	144.89	133.17	150.29	131.18
	IAE	5.04	4.79	5.67	4.68
	ISE	3.62	3.32	3.75	3.27
	ISV	145.98	135.13	151.30	132.23

**Table 5:** Performance index results without noise perturbations.

Methods	Ts/s	OS%	Avg CTs/s
KM	15	0.4	0.0059
WM	15	0.5	0.0058
NT	14	0.3	0.0056
BMM	15	0.4	0.0060

**Table 6:** The parameters of the IT2FPID, PID, and T1FPID controllers.

Methods	K_P_	K_I_	K_D_
IT2FPID	4	0.01	80
T1FPID	1	0.01	45
PID	1	0.01	35

**Table 7:** Performance index results, without noise perturbations.

Methods	Ts/s	OS%	Avg CTs/s
PID	19	20	0.0028
T1FPID	18	50	0.0034
IT2FPID	10	0.3	0.0018

## Data Availability

Not applicable.
